# Application of long short-term memory neural network technique for predicting monthly pan evaporation

**DOI:** 10.1038/s41598-021-99999-y

**Published:** 2021-10-20

**Authors:** Mustafa Abed, Monzur Alam Imteaz, Ali Najah Ahmed, Yuk Feng Huang

**Affiliations:** 1 Department of Civil and Construction Engineering, Swinburne University of Technology, Hawthorn, Melbourne, VIC 3122 Australia; 2 Department of Civil Engineering, College of Engineering, Universiti Tenaga Nasional (UNITEN), Selangor, 43000 Malaysia; 3 Department of Civil Engineering, Lee Kong Chian Faculty of Engineering and Science, Universiti Tunku Abdul Rahman, Selangor, Malaysia

**Keywords:** Hydrology, Engineering

## Abstract

Evaporation is a key element for water resource management, hydrological modelling, and irrigation system designing. Monthly evaporation (Ep) was projected by deploying three machine learning (ML) models included Extreme Gradient Boosting, ElasticNet Linear Regression, and Long Short-Term Memory; and two empirical techniques namely Stephens-Stewart and Thornthwaite. The aim of this study is to develop a reliable generalised model to predict evaporation throughout Malaysia. In this context, monthly meteorological statistics from two weather stations in Malaysia were utilised for training and testing the models on the basis of climatic aspects such as maximum temperature, mean temperature, minimum temperature, wind speed, relative humidity, and solar radiation for the period of 2000–2019. For every approach, multiple models were formulated by utilising various combinations of input parameters and other model factors. The performance of models was assessed by utilising standard statistical measures. The outcomes indicated that the three machine learning models formulated outclassed empirical models and could considerably enhance the precision of monthly Ep estimate even with the same combinations of inputs. In addition, the performance assessment showed that Long Short-Term Memory Neural Network (LSTM) offered the most precise monthly Ep estimations from all the studied models for both stations. The LSTM-10 model performance measures were (R^2^ = 0.970, MAE = 0.135, MSE = 0.027, RMSE = 0.166, RAE = 0.173, RSE = 0.029) for Alor Setar and (R^2^ = 0.986, MAE = 0.058, MSE = 0.005, RMSE = 0.074, RAE = 0.120, RSE = 0.013) for Kota Bharu.

## Introduction

Evaporation is a major constituent of the hydrological cycle and projecting evaporation loss is mainly vital for managing water resources, assessing irrigation scheduling and agricultural modelling^1–4^. Evaporation is impacted by the heat energy supply and vapour pressure gradient, which are mostly reliant on meteorological data like air temperature, solar radiation, relative humidity, wind speed, and atmospheric pressure^5–7^. These aspects are also meticulously associated with other aspects such as geographic location, time of day, current season, and kind of climate. Thus, the procedure of evaporation is extremely non-linear and intricate in nature. Overall, there are two key methodologies, i.e., direct and indirect processes, for computing and estimating evaporation. Direct methods like pan evaporation (Epan) are extensively deployed for estimating evaporation. Notably, it is unfeasible to position pan evaporimeters in all places, particularly in inaccessible regions where precise instrumentation cannot be established or sustained^8^. In an indirect manner, evaporation is projected from empirical equations by utilising other meteorological factors such as maximum and minimum temperature, wind speed, sunshine hours, and relative humidity. Precise gauging of such meteorological factors is a tedious task and entails different sophisticated tools and skilled labour force^9^. Frequently, instrument fault, inappropriate operation and upkeep, and hostile weather conditions make it tough to gauge these data minus any mistakes, which is vital for projecting evaporation through empirical equations. Any mistake in gauging these factors would cause considerable direct concerns in projecting evaporation.

Hence, indirect techniques of projecting evaporation by utilising empirical equations are a data-sensitive procedure and also guided by different presumptions. Furthermore, the availability of all such meteorological data at a particular weather station is scarce or not easily available and generally discontinuous in certain areas^10^. Because of the extremely intricate physical and nonlinear form of the evaporation procedure, it is tough to model evaporation by means of empirical techniques^11^. Furthermore, an empirical model formulated for one agro-climatic scenario might not perform fine in other circumstances and entails recalibration of model coefficients prior to execution^12^. Earlier, few attempts were made by academics to model the evaporation procedure by formulating many empirical formulae, which are mentioned in the literature^13^. The selection of optimal model inputs has always been a challenge for the non-linear regression process, and several studies have shown that evaporation is influenced by input weather variables such as air temperature, relative humidity, solar radiation, and wind speed^14^. Thus, developing accurate empirical models to represent all these complex processes is difficult^15^.

## Literature review

Of late, artificial intelligence (AI) based soft computing methods such as support vector machines (SVM), adaptive neuro-fuzzy inference system (ANFIS), M5 model tree (MT), artificial neural network (ANN), gene expression programming (GEP) and extreme learning machine (ELM) have been effectively deployed for dealing with an extensive gamut of ecological and water engineering issues^14,16–20^. Artificial intelligence approaches are easier, vigorous and able to deal with the intricate non-linear procedures without difficulty^8,21,22^. Several studies were recorded about utilising the AI techniques for forecasting diverse hydrological procedures^23^. They noted that ANN models offer superior estimates as against the traditional techniques. For instance, Castellano-Méndez et al.^24^ made a comparison of ANN and Box & Jenkins methodologies and deduced that ANN is an upgrade on the Box & Jenkins model regarding the simulation of prospective runoffs with high level of precision.

With regards to evaporation projection and taking into account the drawbacks related to both empirical and measurement methods discussed until now, various studies have also been carried out by utilising machine learning (ML) methodologies with diverse optimization algorithms for estimating pan evaporation^25,26^. These have offered certain substitute machine learning solutions to the issue with diverse input combinations of existing climatic variables like humidity, temperature, wind speed, solar radiation, sunshine, and vapour pressure^27,28^. Keskin and Terzi^29^ employed the ANN and Penman models for modelling evaporation. They utilised many meteorological factors as inputs for the ANNs. The researchers noted that as against the Penman model, ANN is superior when it comes to projecting evaporation. Kişi^30^ deployed evolutionary neural networks for projecting monthly pan evaporation. The outcomes showed that the recommended models offered superior precision over the empirical techniques. Wang et al.^31^ studied the ability of generalized regression neural network (GRNN), multilayer perceptron (MLP), least square support vector machine (LSSVM), fuzzy genetic (FG), multivariate adaptive regression spline (MARS) and adaptive neuro-fuzzy inference systems with grid partition (ANFIS-GP) for projecting evaporation. They made a comparison of the outcomes with regression methods in various climatic scenarios of China. The researchers noted that heuristic methods usually delivered superior performance compared to empirical and regression methods.

In a research conducted by Deo et al.^32^, monthly evaporative losses were projected by utilising three machine learning approaches, namely Extreme Learning Machine (ELM), Relevance Vector Machine (RVM), and Multivariate Adaptive Regression Spline. Meteorological factors were utilised as the predictor variable and RVM was observed to be the best approach out of these. Sudheer et al.^21^ deployed an ANN model for patterning daily evaporation and noted that the ANN model can be utilised effectively for projecting the evaporation rate based on climate data. Falamarzi et al.^33^ studied the usage of ANN and wavelet ANN for patterning daily evaporation. They utilised wind speed and temperature data as inputs for the models. The outcomes showed that both models precisely projected evaporation. Wang et al.^31^ projected daily Ep by utilising least square support vector regression (LSSVR), fuzzy genetic (FG), multivariate adaptive regression spline (MARS), M5 model tree (M5Tree) and multiple linear regression (MLR) for eight stations around China’s Dongting Lake basin. Studies indicate that LSSVR and FG offer superior performance compared to other machine learning methods. Monthly EP was projected by Malik et al.^34^ in the Indian central Himalayas area by deploying MLPNN, radial basis neural network (RBNN), co-active neuro-fuzzy inference system (CANFIS), and self-organizing map neural network (SOMNN). Gamma test was utilised for choosing apt input combination. The researchers noted the dominance of CANFIS over other methodologies. Tezel and Buyukyildiz^35^ scrutinised the usage of RBFN, MLP, and e-support vector regression (SVR) by utilising diverse training algorithms. Both SVR and ANNs with a scaled conjugate gradient (SCG) learning delivered better performance in comparison to empirical approaches. In Turkey, Kisi et al.^36^ studied the ability of decision tree-based machine learning methods like Chi-square automatic interaction detector (CHAID) and classification and regression tree (CART) and compared them with the neural network model for day-to-day EP projection. The outcomes indicated that neural networks delivered better performance in comparison to other models in various circumstances.

However, most of these studies chiefly focused on exploring the generalised abilities of ML models in various weathers because every climate has its own features of non-stationarity and stochasticity. ML models like the CART (classification and regression tree), the SVM (support vector machine), the CCNN (cascade correlation neural network), and GEP (gene expression programming) have achieved noteworthy advancements in hydrologic modelling^37–41^. These models can proficiently simulate and explain the stochasticity of various complex hydro-climatological activities. Recent evaporation forecast studies have revealed a noticeable success through better, more consistent generalised predictive models. It has also been the purpose when forming and implementing novel evaporation prediction techniques, since the target is to attain low prediction errors. It is ascertained from the review of the literature that ANNs with suitable learning algorithms are confirmed to be potentially able to model the evaporation process in different locations and have achieved better results than more complex traditional frameworks^42^. The task of prediction is nonlinear in nature, and thus the adaptive prediction model should have nonlinear aspects. Nevertheless, the selection and formation of efficient, impressive, and reliable techniques for accurately predicting evaporation remain difficult for the experts as evaporation is complex in nature and a greatly nonlinear process.

In recent times, the American researcher Chen and Guestrin^43^ presented a new powerful learning model called XGBoost, which is extensively used by data experts and has obtained state-of-the-art outcomes in several areas because of its general scalability. For instance, Lei et al.^44^ used 6 machine learning approaches to create the prediction models, and the XGBoost model achieved the most precise qualitative predictions. Nonetheless, amongst various techniques known for a long time, one deep learning method of ANN, known as LSTM-NN (Long Short-Term Memory Neural Networks), has drawn attention for time series prediction^45^. The estimations by this class are affected by the system’s previous behaviour, and it can be employed for both classification and regression purposes. In comparison to other deep models, like the deep Boltzman machine, convolutional neural networks (CNN), and graph-structured recurrent neural network, the LSTM-NN deep learning models perform significantly better. Through a specifically designed architecture, LSTM-NNs have shown better modelling capabilities in different time series applications. In more recent years, Zhang et al.^46^ related the performance of various neural network models for simulating and forecasting the water levels of an integrated sewer structure in Norway, on the basis of online data from water-level sensors and rain gauges. They proved that LSTM is better adapted for multi-step-ahead estimations than traditional models without explicit cell memory. Zhang et al.^46^ employed an LSTM model for estimating water tables in agricultural regions. In addition, the authors compared the resultant model from the LSTM-based method with that of a conventional neural network and discovered that the former performs better than the latter.

## Objectives

This study’s contribution is to examine the capability and establish the efficiency of the LSTM (Long Short-Term Memory) neural networks in forecasting monthly evaporation (Ep) using information from two climatological stations located in Malaysia: Kota Bharu and Alor Setar. The performance of LSTM was compared with the Extreme Gradient Boosting (Xgboost) as the most reliable ML model and with the ElasticNet Linear Regression (LR). Moreover, the recently developed ML models were compared with two empirical techniques, namely Stephens & Stewart and Thornthwaite. After predicting Ep, the efficiencies of the proposed models were examined and evaluated using various selected performance parameters to assess their effectiveness in the field of evaporation forecasting. Furthermore, the application of the proposed methodology to various regions throughout Malaysia using different data sets will also be investigated in the future work.

## Methodology

### Study area and data collection

**Figure 1:**
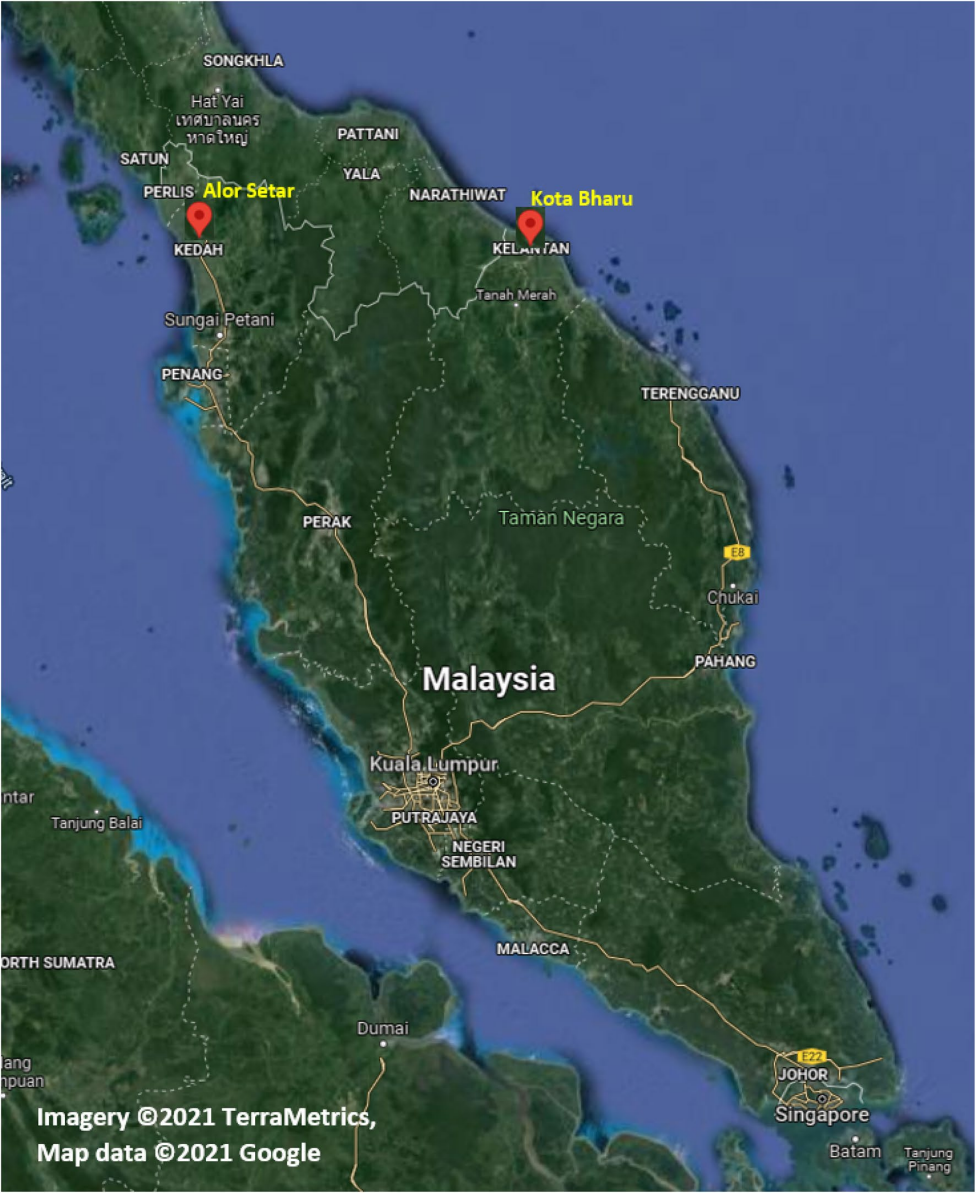
Location of case study [Imagery ©2021 TerraMetrics, Map data ©2021 Google].

Malaysia is a tropical country with a high rate of rainfall. However, as a result of development, there is an increase in water demand. Furthermore, global climate change lengthens the dry season and increases the rate of evaporation from impounding reservoirs. This research aims to develop a reliable generalised model to predict evaporation throughout Malaysia. The relevance of the models for evaporation prediction was investigated in Kota Bharu and Alor Setar meteorological stations as part of this research. The climate data from two stations, Alor Setar (longitude 100° 24′ E, latitude 6° 12′ N, elevation 3.4 m) as well as Kota Bharu (longitude 102° 18′ E, latitude 6° 10′ N, elevation 4.4 m), managed by the MMD (Malaysian Meteorological Department), are used in this study. Figure 1 displays the location of these stations on the map of Malaysia. Figure 1 has been generated by using Google Map software to identify the location of the study area.

**Figure 2:**
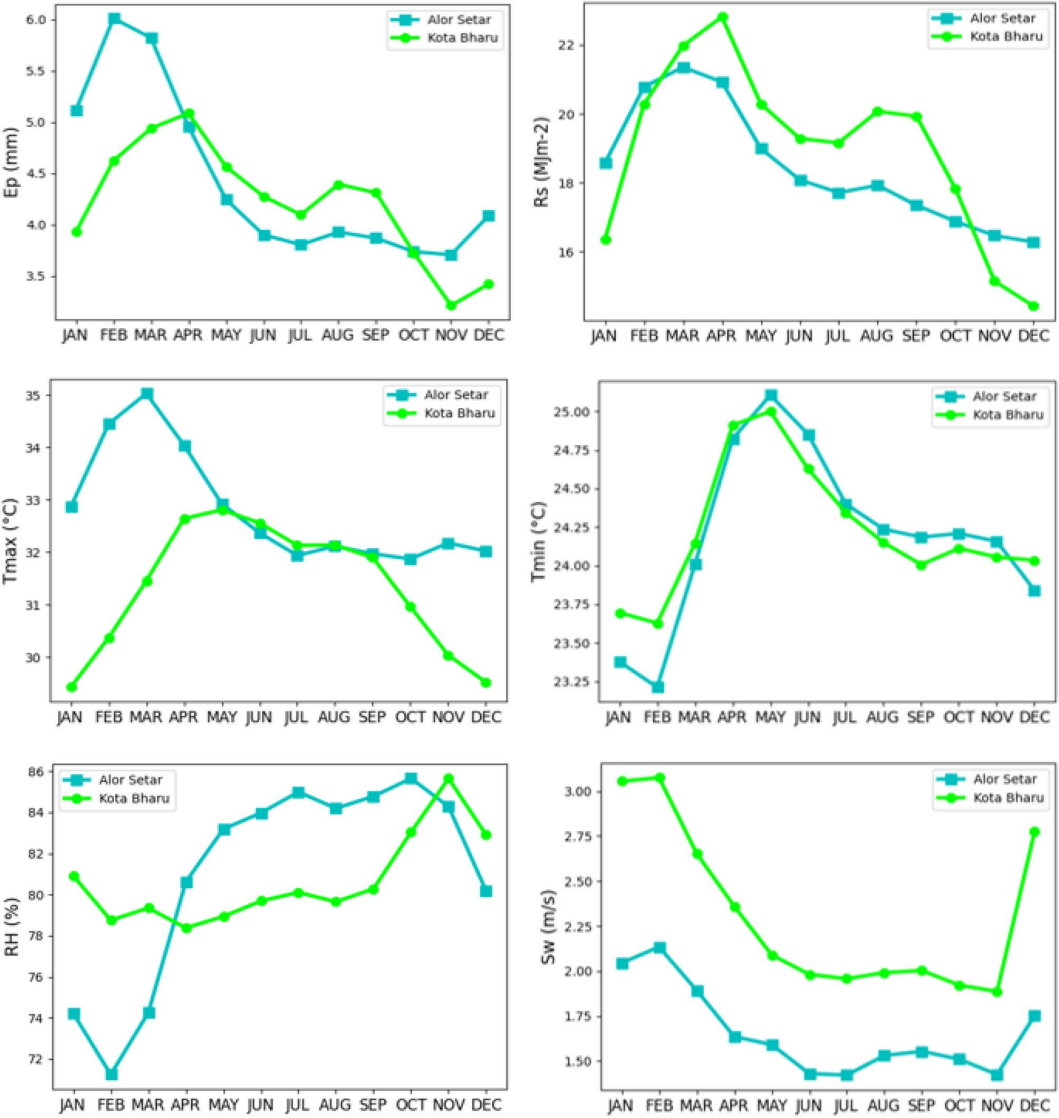
Monthly variations of Ep and associated meteorological parameters used in this study.

Weather parameters measured include minimum, maximum and mean air temperature (*T*_*min*_, *T*_*max*_, *T*_*a*_), wind speed (*S*_*w*_), relative humidity (*RH*), open pan evaporation (*E*_*p*_) and solar radiation (*R*_*s*_). The data set comprised 19 years of daily reports from 2000 to 2019. Statistical parameters regarding the climatic data are shown in Table 1 and Fig. 2.

**Table 1:** Various meteorological variables and their descriptive statistics.

Station	Dataset	Unit	*X*_*mean*_	*S*_*x*_	*C*_*v*_	*C*_*x*_	*X*_*min*_	*X*_*max*_
Alor Setar	*T*_*max*_	°C	32.81	1.82	5.55	− 0.35	24.8	39.1
	*T*_*min*_	°C	24.19	1.06	4.38	− 0.36	19.2	27.7
	*RH*	%	80.91	7.38	9.12	− 0.98	49.8	96.8
	*S*_*w*_	m/s	1.66	0.57	34.33	0.59	0.1	4.7
	*R*_*s*_	MJ m^−2^	18.44	4.72	25.59	− 0.81	0.66	27.69
	*E*_*p*_	mm	4.43	1.59	35.85	0.31	0.1	9.9
Kota Bharu	*T*_*max*_	°C	31.34	1.64	5.23	− 0.56	24.4	35.9
	*T*_*min*_	°C	24.22	1.06	4.41	− 0.33	17.8	27.9
	*RH*	%	80.60	4.66	5.79	0.38	61.9	98.3
	*S*_*w*_	m/s	2.31	0.90	39.03	1.99	0.4	8.6
	*R*_*s*_	MJ m^−2^	19	5.25	27.65	− 0.87	0.6	28.9
	*E*_*p*_	mm	4.22	1.32	31.35	− 0.22	0	9.5

In this table, the *X*_*mean*_*, S*_*x*_*, C*_*v*_*, C*_*x*_*, X*_*max*_ and *X*_*min*_ represent the mean, standard deviation, coefficient of variation, skewness, maximum and minimum of the weather variables, respectively.

As far as the climate is concerned, in Alor Setar region, the summers are hot and short; the winters are warm, long, and wet; and it is gloomy and oppressive year-round. During the year, the temperature usually varies from 23 to 34 °C and is seldom less than 22 °C or more than 36 °C. The hot weather lasts for 1.9 months, beginning in February and ending in April, with a mean daily high temperature more than 33 °C. The winter lasts for 6 months, beginning in June and ending in December, with a mean daily high temperature less than 31 °C. The average part of the sky cloaked by clouds experiences noteworthy seasonal variation during the year. In Alor Setar, the sunnier part of the year lasts for 3.6 months beginning in December and ending around March. The clearest day is the mid of January when the sky remains clear, mostly sunny, or partially cloudy for 35% of the time, and mostly cloudy or gloomy for 65% time.

The summer season in Kota Bharu is hot, while the winter season is warm but relatively short; the area witnesses cloudy sky throughout the year. Annual temperatures range between 23 and 32 °C; temperatures are rarely above 34 °C or below 21 °C. The average hot summer duration is 3.2 months, covering April, May, June, and July. On the other hand, November, December, January, and February are relatively cool, with an average duration of 2.6 months; the average high temperature remains less than 29 °C. There is a noteworthy difference in cloud cover over seasons. Kota Bharu skies are clearer for 3.9 months beginning in December and ending in April. The sky is clearest in February, which has the clearest day of the year. Additionally, there is a 36% incidence of clear, partially clear, or partly cloudy sky, while higher cloudiness is present for the remaining 64% of the month.

### Selection of input combinations and data partitioning

**Figure 3:**
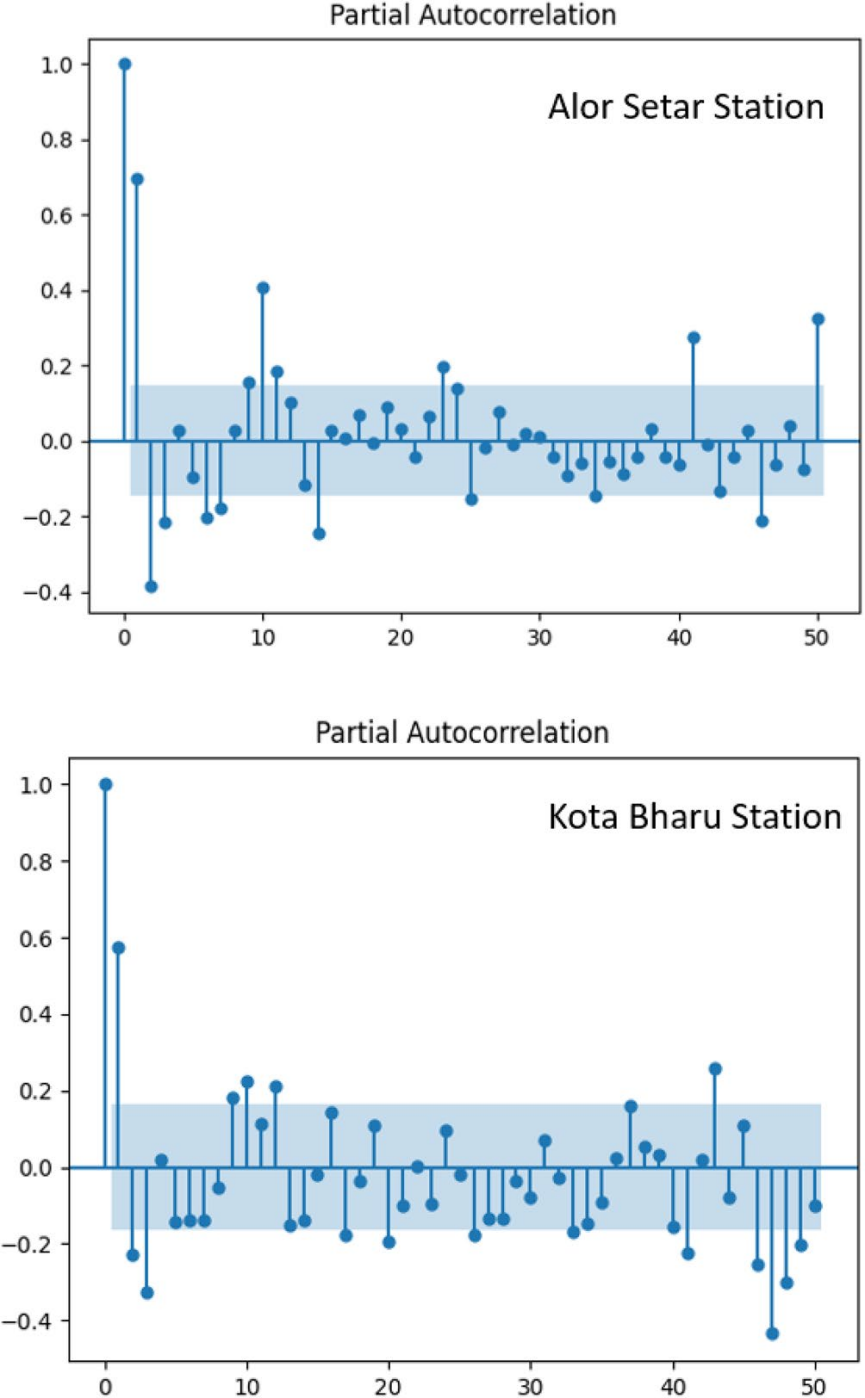
Partial Autocorrelation for Alor Setar and Kota Bharu stations (Monthly).

Input variability can substantially affect the modelling procedure and prediction accuracy; therefore, different combinations of input data were explored in the present study to ascertain optimal ML model predictability for evaporation. There are specific conscious choices for selecting these combinations. First, for comparison purpose, input parameters to the machine learning models (XGR, ElasticNet LR, and LSTM) were chosen based on the needed meteorological aspects in the two proposed empirical models (Stephens–Stewart and Thornthwaite). Second, availability of consistent long-term weather data has always been one of the major constraints in deciding on input combination. In fact, one or two missing meteorological parameters for model inputs can be expected in some case studies, primarily in developing countries. It is advantageous then to investigate the level of prediction accuracy of ML models in the absence of any input parameters. This will also provide a better practical understanding of how each input variable affects the evaporation forecast in that region. Hence, one of the objectives of this study is to explore the predictive capability of ML models under various input combinations of meteorological variables to successfully map the model input–output with a high level of prediction accuracy. Moreover, the present research assesses the effects of input variable E_p._ In this context, the input data records were chosen based on how the antecedent records were related to the predicted output value. As shown in Fig. 3, the autocorrelation analysis for the historical monthly time series for the pan evaporation rate revealed that the correlation deteriorated significantly once it passed the preceding second lag-time record. This demonstrates that the preceding second evaporation rate record influenced the evaporation rate at any time. Consequently, the maximum lag time of two antecedent records was used as the model input when developing the proposed model for the monthly time increment. Table 2 depicted the ten combinations of inputs having varying T_min_, T_max_, T_a_, R_s,_ S_w_, RH and E_p._

**Table 2:** Input combinations of meteorological variables used for machine learning models.

No.	Model			Input combinations
	ElasticNet LR	XGB	LSTM	
1	ElasticNet LR-1	XGB-1	LSTM-1	T_a_
2	ElasticNet LR-2	XGB-2	LSTM-2	T_max_, T_min_
3	ElasticNet LR-3	XGB-3	LSTM-3	T_max_, T_min_, RH
4	ElasticNet LR-4	XGB-4	LSTM-4	T_max_, T_min_, RH,S_w_
5	ElasticNet LR-5	XGB-5	LSTM-5	T_max_, T_min_, RH, R_S_
6	ElasticNet LR-6	XGB-6	LSTM-6	T_a,_ R_S_
7	ElasticNet LR-7	XGB-7	LSTM-7	T_max_, T_min_, R_s_
8	ElasticNet LR-8	XGB-8	LSTM-8	T_max_, T_min_, R_s_, S_w_
9	ElasticNet LR-9	XGB-9	LSTM-9	T_max_, T_min_, R_s_, S_w_, RH
10	ElasticNet LR-10	XGB-10	LSTM-10	T_max_, T_min_, R_s_, S_w_, RH, E_p_

The 80/20 principle was used to split the quantified meteorological data into training, and testing sets required for ML modelling; 80% of data were employed for model training, while the remaining 20% were used for testing. The training set was used to initiate ML parameter training. Subsequently, the test set was employed to assess the model. Thus, the dataset was divided by taking the first years for training and the last ones for testing. The present study aims to perform a detailed evaluation for testing soft computing capability and using practical frameworks for predicting monthly evaporation levels at the Kota Bharu and Alor Setar regions.

### Empirical models used for monthly pan evaporation prediction

Taking into consideration the number of required meteorological inputs and data availability, two empirical techniques, namely Stephens–Stewart and Thornthwaite, have been selected in this study as the most commonly used methods^47^.

### Stephens and Stewart

This method also called the “Fractional Evaporation-Equivalent of Solar Energy” by Stephens and Stewart^48^. Stephens and Stewart suggested that using measured radiation Qs, give a better result where data are available and can be correlated with temperature, as shown in Eq. (1):

1 $$Ep= \left(0.0082Ta-0.19\right)\left(\frac{Qs}{1500}\right) \times 25.4,$$

where $Ta$, $Ep$, and $Qs$ denotes mean air temperature (F), evaporation (mm), and solar radiation (cal cm^−2^ day^−1^). Stephens and Stewart also suggested the desirability of continued studies in other regions to establish such relations under a range of climatic conditions.

### Thornthwaite

Thornthwaite^49^ used practical data to ascertain the correlation between probable evaporation ($Ep$) and mean monthly temperature ($Ta$) and standardised it to a 30-day month, comprising 12 h of sunshine per day. The Thornthwaite technique is used to compute potential evaporation ($Ep$); the following expression is used to compute the Monthly Thornthwaite Heat Index ($i$):

2 $$i = \left( {\frac{{Ta}}{5}} \right)^{{1.514}} ,$$

where $Ta$ denote mean monthly temperature (°C).

The Annual heat index $\left(I\right)$ is the aggregate of Monthly Heat Indices $\left(i\right)$:

3 $$I=\sum_{i=1}^{12}i.$$

A potential Evaporation $Ep$ estimation is obtained for each month by applying the following equation:

4 $$Ep=16\times {\left(\frac{10. Ta}{I}\right)}^{a},$$

where $$a$$ is:

5 $$a=\left(675 \times {10}^{-9} \times {I}^{3}\right)-\left(771 \times {10}^{-7}\times {I}^{3}\right)-\left(1792\times {10}^{-5}\times I\right)+0.49239.$$

$Ep$ for a given month is, therefore, given by the expression:

6 $$Ep={Ep}_{Obtained} \cdot \frac{N}{12}\cdot \frac{d}{30} \; \left(\text{mm}\right).$$

$N$ and *d* denote theoretical monthly sunshine hours, and days in the month, respectively.

### Machine learning (ML) models used for monthly pan evaporation prediction

The present study used three ML frameworks for estimating evaporation. These models are Extreme Gradient Boosting (XGB)^43^, ElasticNet Linear Regression (ElasticNet LR)^50^, and Long Short-Term Memory (LSTM)^45^. The training and testing for the machine learning models were carried out by using the TensorFlow framework on an NVIDIA GeForce GTX 1080 Ti GPU.

#### ElasticNet linear regression

ElasticNet LR is a regularisation method for linear regressions. It is typically employed for addressing linear regression overfitting^50–52^. This method applies the elastic net penalty on the least-squares calculation. The method comprises two widely-used penalty expressions (L1 and L2); these are included with the loss function while the system is trained^50,51^. The method allows addressing the challenges concerning the lasso method. The ElasticNet technique combines the advantages of the Ridge Regression and Lasso method, thereby creating a trade-off between the constituent methods.

7 $$\beta^{\prime} = \arg \min_{\beta } \left( {\left\| {y - X\beta } \right\|^{2} + \lambda_{2} \left\| \beta \right\|^{2} + \lambda_{1} \left\| \beta \right\|_{1} } \right),$$

β′ denote the ideal weights required for minimising the loss function, which comprises the squared difference of the real and predicted values, including the two regularisation items. Penalty expressions for L1 and L2 are λ1 ‖β‖_1_, and λ2 ‖β‖^2^; here, λ coefficients must be tuned.

Both alpha and l1_ratio was tuned to select the optimal values. Alpha with values [0.001, 0.01, 0.1, 1, 10, 100, 1000] and l1_ratio with values [0, 0.1, 0.2, 0.3, 0.4, 0.5, 0.6, 0.7, 0.8, 0.9, 1] were evaluated. A constant value, alpha, is used for penalty term multiplication; l1_ratio penalty as an L2 type penalty with zero value; L1 penalty for l1_ratio = 1; in case l1_ratio is between 0 and 1, a combined L1 and L2 penalty is applied.

#### Extreme gradient boosting (XGB)

XGB is a scalable end-to-end tree-based learning framework with more than ten times the speed of present offerings meant for a single machine. XGB scaling comprises numerous examples for memory-restricted applications^43,50^. XGB scalability is the consequence of numerous optimisations implemented for addressing the roadblock. This framework employs the gradient descent technique for loss minimisation, while regularisation is used to regulate overfitting.

In our experiment, we try different set of values of hyperparameters to select the optimal one which is given as follows:

1. Least squares regression Loss function to be optimized.
2. Learning rate = 0.5.
3. Number of estimators = 100; gradient boosting is quite robust to over-fitting, and hence a large number typically drives superior performance.
4. The number of features to consider when looking for the best split max_features = ’‘sqrt’ “sqrt”, then max_features = sqrt(n_features).
5. Max_depth = 3; the maximum depth limits the number of nodes in the tree.

#### Long short-term memory (LSTM)

**Figure 4:**
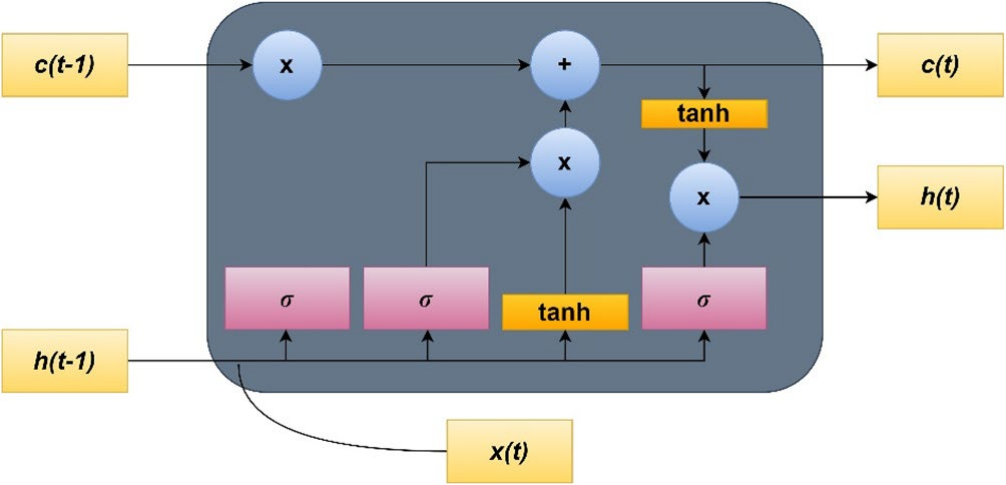
LSTM neural network cell.

LSTM belongs to the Recurrent Neural Network (RNN) category and is employed for long-range sequence models. Figure 4 depicts an LSTM memory cell that store state information and is regulated using gates. This system reduces gradient vanishing. An LSTM structure can record temporal associations.

The present study applies several inputs like humidity, temperature, wind, evaporation, and solar radiation to LSTM. Iterative LSTM parameter tuning was performed for data fitting. Several hyperparameters were evaluated to identify the optimal LSTM architecture that provides ideal assessment metrics.

The hyperparameters include:

1. number of LSTM layers;
2. number of nodes in each layer;
3. number of fully connected layers;
4. types of activation function;
5. number of dropout layers and percentage of dropout;
6. learning rate;
7. loss function;
8. optimizer;
9. batch size;
10. number of epochs.

The ideal LSTM structure for the study comprises these layers:

1. LSTM with 512 nodes.
2. Fully connected layers with 64 nodes and ReLU activation function
3. Dropout with 0.4%.
4. Fully connected layers with 1 node and Linear activation function.

The final hyperparameters are:

1. the learning rate is 0.001;
2. loss function of mean absolute error (MAE);
3. optimizer: ADAM;
4. epochs is 500;
5. the batch size is 8.

In general, AI models are chosen based on the availability of data and their ability to solve the targeted problem. There are various benefits and drawbacks for each model that have been observed across all AI models. Table 3 summarises the advantages and disadvantages of each model. However, since each model behaves separately depending on the problem it was designed to solve, these benefits and drawbacks may not be necessarily lined with the model.

**Table 3:** Advantages and disadvantages of the proposed machine learning models.

AI model type	Advantages	Disadvantages
ElasticNet LR	ElasticNet is a Lasso/Ridge hybrid that benefits from both the L1 (Lasso) and L2 (Ridge) regularizers Simple model and can be regularised to avoid overfitting Performs well when there are several features that are related to one another	Performs poorly when there are non-linear relationships since they are not naturally flexible enough to capture more complex patterns, and adding the appropriate interaction terms or polynomials can be difficult and time-consuming
XGB	Boosting is a persistence and robust method for preventing and mitigating over-fitting Flexible to adapt Extremely fast computation	High-sensitivity to outliers Does not perform very well on large data sets
LSTM	Ability to learn extremely complicated patterns Ability to generate new features from limited set of training data, and to easily update them with new data Powerful deep learning algorithm that is able to model complicated and highly non-linear processes without any constraints on the input–output vector relationships	Tuning requires a high level of expertise (i.e. set the architecture and hyperparameters) High-speed processing units and powerful GPUs are required for training the data sets

### Performance evaluation

**Figure 5:**
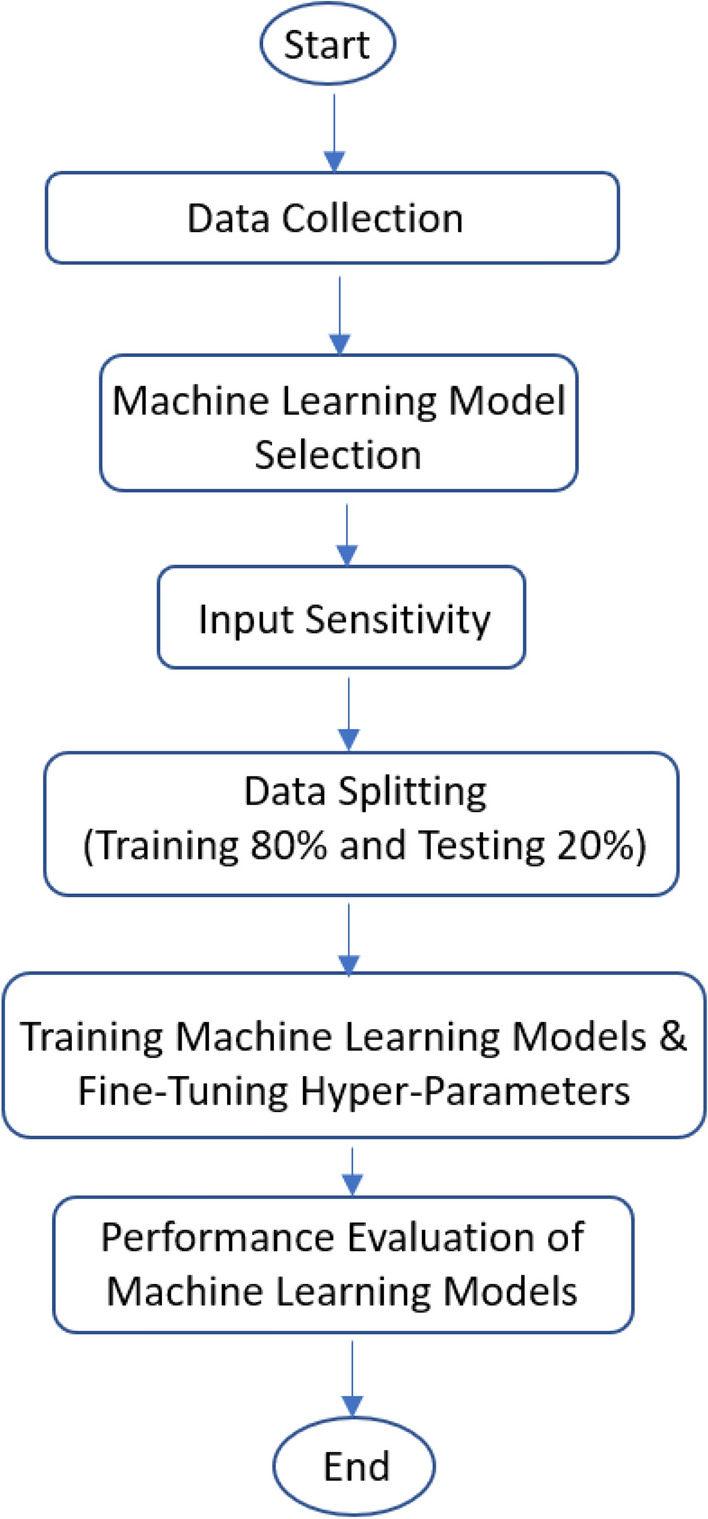
Flow chart of the proposed methodology to forecast evaporation using machine learning models.

Model predictive performance was assessed using several statistical indicators like mean absolute error (MSE), determination coefficient (R^2^), relative absolute error (RAE), root mean square error (RMSE), relative squared error (RSE) and mean absolute error (MAE). These indicators are described below:

1. The determination coefficient (R^2^) is an indicator that specifies the correlation between the real and predicted output; it lies between zero and one (both inclusive). Zero value indicates a random model, while one indicates a perfect fit.
  8 $${R}^{2}= \frac{\sum_{i=1}^{n}\left(y- \overline{y }\right) (\widehat{y}- \overline{\widehat{y} })}{\sqrt{\sum_{i=1}^{n}{(y- \overline{y })}^{2 } } \sum_{i=1}^{n}{(\widehat{y}- \overline{\widehat{y} })}^{2} }.$$
2. *Mean absolute error (MAE)* the absolute error existing between the real and predicted output.
  9 $$MAE= \frac{1}{n}\sum_{i=1}^{n}\left|y- \widehat{y}\right|.$$
3. *Mean square error (MSE)* the average squared error existing between the predicted and real output.
  10 $$MSE= \frac{1}{n} {\sum_{i=1}^{n}(y- \widehat{y})^{2}}.$$
4. *Root mean square error (RMSE)* the square root of average squared error existing between the predicted and real output.
  11 $$RMSE=\sqrt{\frac{{\sum_{i=1}^{n}(y- \widehat{y})}^{2}}{n}}.$$
5. *Relative absolute error (RAE)* The magnitudes of real and predicted outputs are subtracted, and the values are aggregated and normalised.
  12 $$RAE= \frac{\sum_{i=1}^{n}\left|y- \widehat{y}\right|}{\sum_{i=1}^{n}\left|y- \overline{y }\right|}.$$
6. *Relative squared error (RSE)* It denotes the normalised aggregate of the squared difference between the predicted and real output.
  13 $$RSE= \frac{\sum_{i=1}^{n}{(y- \widehat{y})}^{2}}{\sum_{i=1}^{n}{(y- \overline{y })}^{2}},$$

where n is the number of samples, y is an actual output, $\widehat{y}$ is a is forecast output, $\overline{y }$ is the average of actual output. To conclude the training and performance evaluation processes, a flow chart has been designed which is shown in Fig. 5. The step-by-step process used in this methodology has been demonstrated in the flow chart.

## Results and discussion

### Estimation of monthly pan evaporation using two empirical models

**Figure 6:**
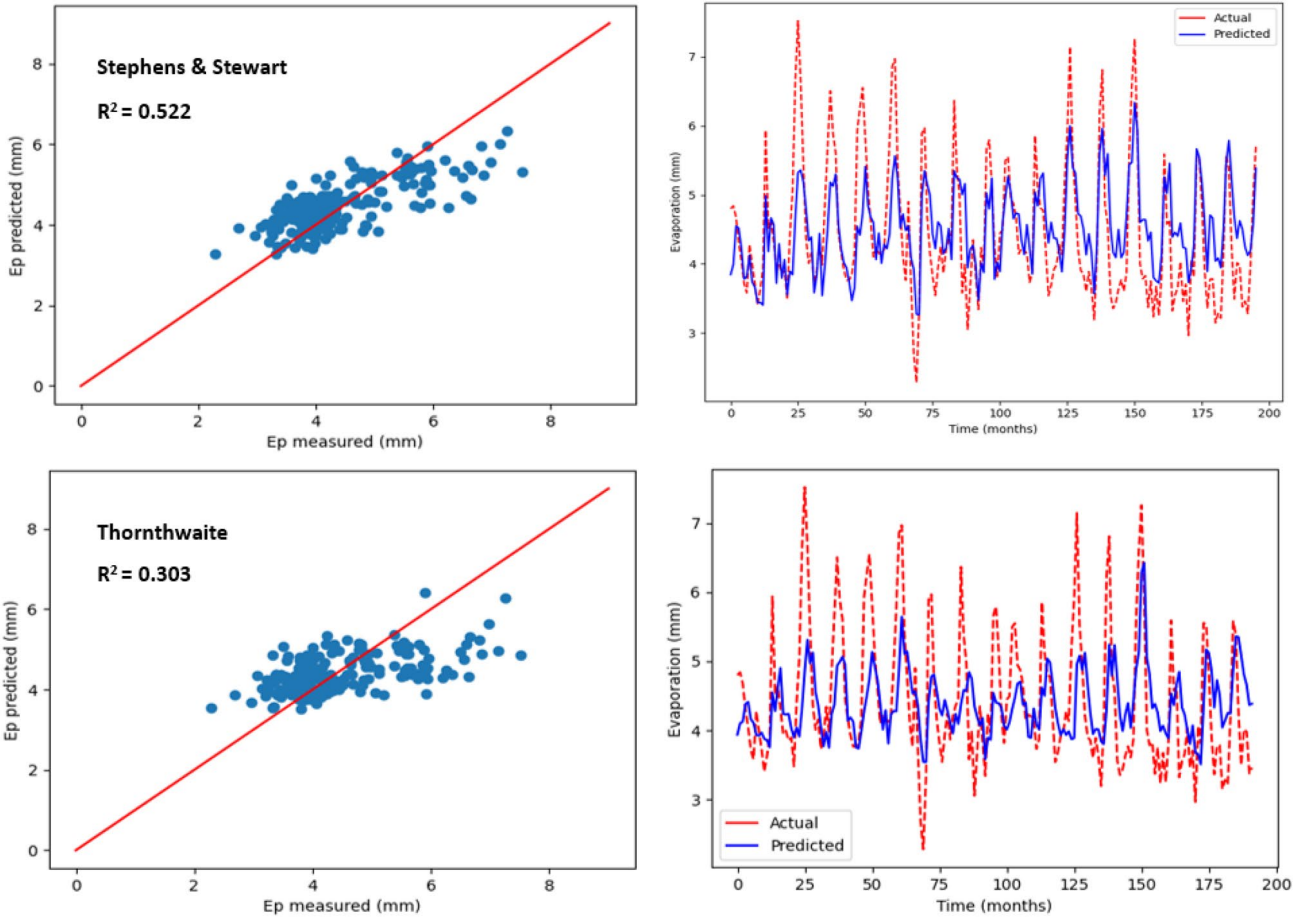
Scatter plot of measured Ep versus predicted Ep for the proposed empirical modles for Alor Setar station.

**Figure 7:**
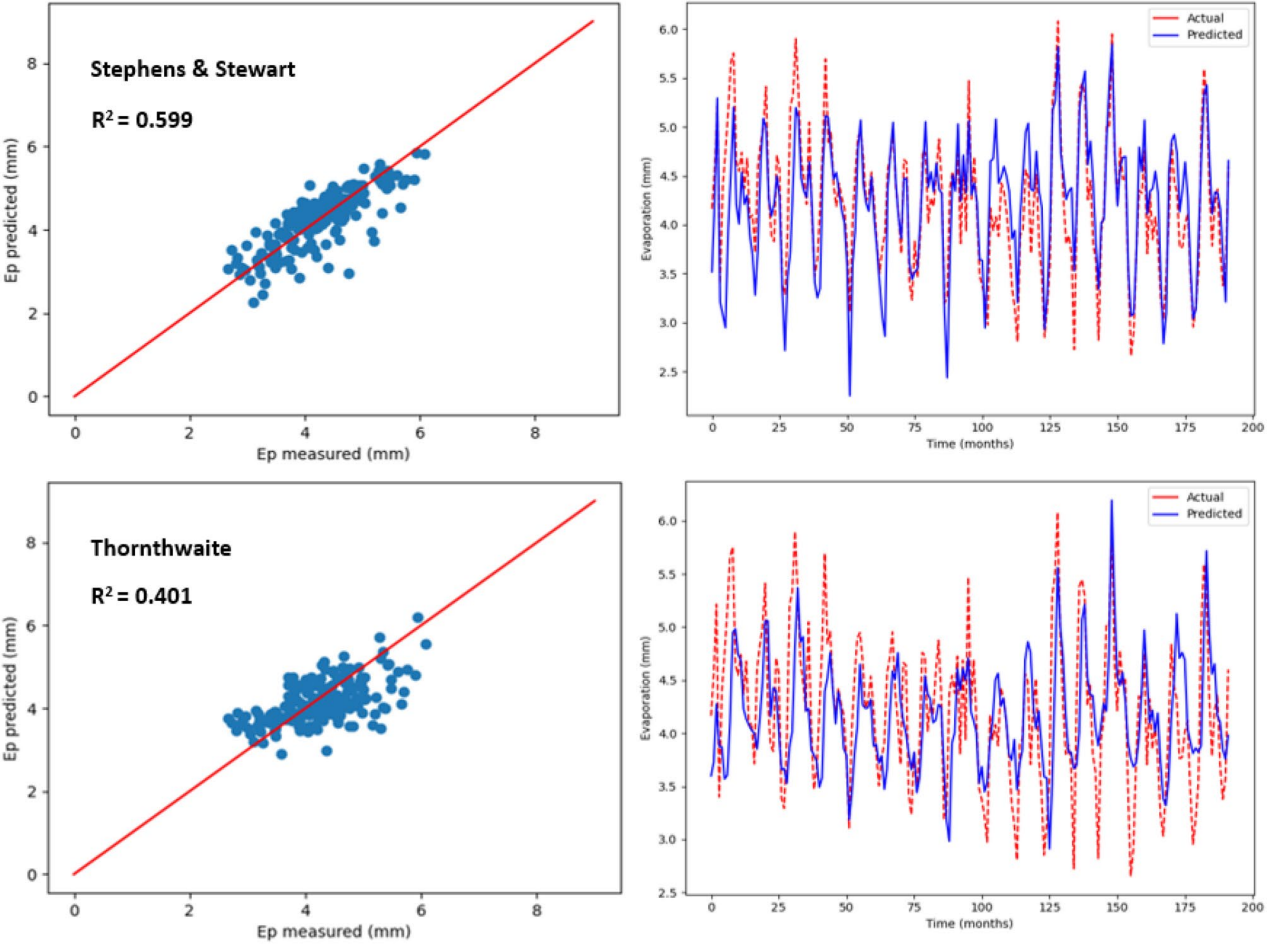
Scatter plot of measured Ep versus predicted Ep for the proposed empirical modles for Kota Bharu station.

As stated earlier, monthly Ep, including the temperature-based and radiation-based models, was predicted by employing two empirical models. The values pertaining to R^2^, MSE, MAE, RSE, RAE and RMSE are listed in Table 4, with regards to the two empirical models employed to predict Ep in Kota Bharu and Alor Setar stations. As suggested by the statistical values listed in Table 4, higher prediction accuracy was observed with the radiation-based model (Stephens and Stewart) compared with the other empirical model. In particular, the highest R^2^ values (0.522 and 0.599) and the least RMSE values (0.677 and 0.436) were seen in the Stephens and Stewart model. While, in the Thornthwaite model, RMSE increased by almost 14%, and the corresponding R^2^ decreased by around 22%. In addition, the lowest MAE (0.535 and 0.33), MSE (0.458 and 0.19), RAE (0.681 and 0.603) and RSE (0.477 and 0.4) values were observed using the Stephens and Stewart model which clearly indicates that Stephens and Stewart model outperformed Thornthwaite model. This could be due to solar radiation inclusion, which normally includes an enhancement over a strictly temperature-based estimate. In Figs. 6 and 7, predicted values pertaining to monthly Ep with regards to the two empirical models have been plotted versus the measured values for Alor Setar and Kota Bharu stations, respectively.

**Table 4:** Statistical results of Stephens and Stewart and Thornthwaite empirical models for prediction Ep at Alor Setar and Kota Bharu stations.

Station	Model	R^2^	MAE	MSE	RMSE	RAE	RSE
Alor Setar	Stephens and Stewart	0.522	0.535	0.458	0.677	0.681	0.477
	Thornthwaite	0.303	0.635	0.67	0.819	0.811	0.696
Kota Bharu	Stephens and Stewart	0.599	0.33	0.19	0.436	0.603	0.400
	Thornthwaite	0.401	0.449	0.33	0.574	0.82	0.693

### Estimation of monthly pan evaporation using three machine learning models

**Figure 8:**
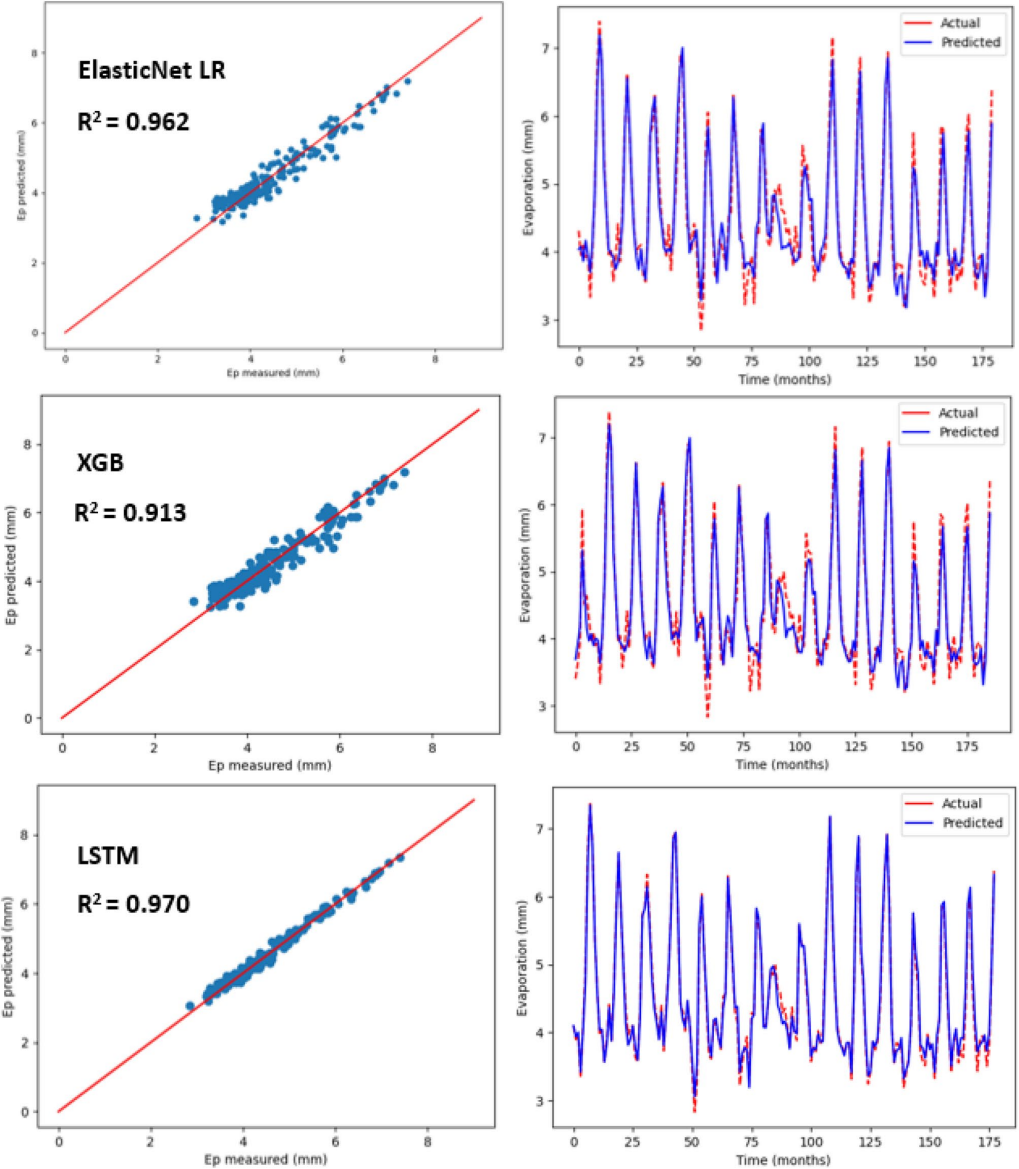
Scatter plot of measured Ep versus predicted Ep for the proposed machine learning models for Alor Setar station.

**Figure 9:**
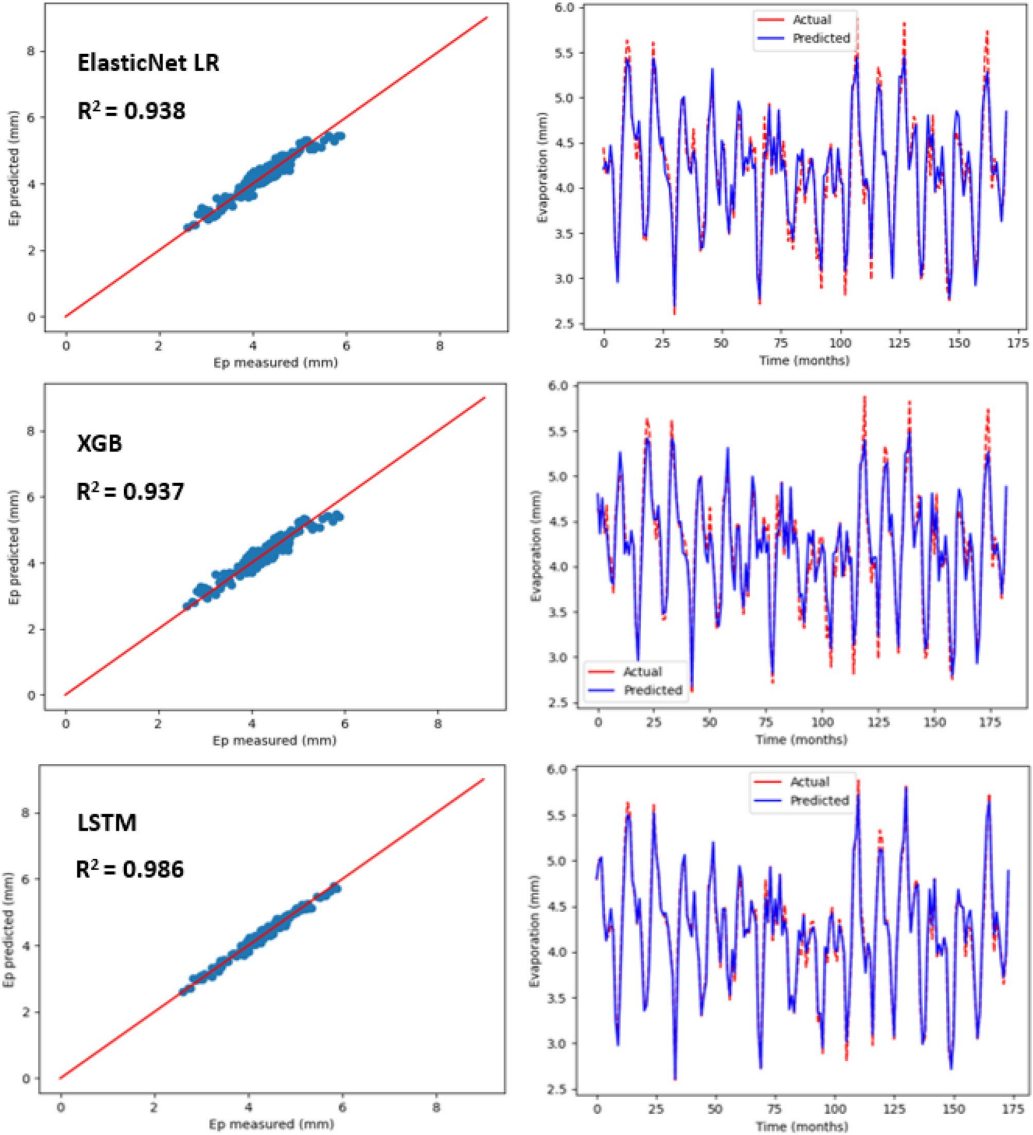
Scatter plot of measured Ep versus predicted Ep for the proposed machine learning models for Kota Bharu station.

Table 5 shows the statistical results pertaining to three machine learning models in order to predict monthly Ep that is placed under 10 input combinations with regards to meteorological variables for Kota Bharu and Alor Setar stations. For each of the machine learning models, the best statistical indicators have been represented in bold. As observed in Table 5, there was a considerable difference in the prediction accuracy of monthly E_p_ based on the input combination as well as model type. As per the statistical values, under various input combinations, with regards to the three machine learning models, the LSTM model (R^2^ = , 0.970, MAE = 0.135, MSE = 0.027, RMSE = 0.166, RAE = 0.173, RSE = 0.029) at the Alor Setar station and (R^2^ = 0.986, MAE = 0.058, MSE = 0.005, RMSE = 0.074, RAE = 0.120, RSE = 0.013) at the Kota Bharu station performed much better than the ElasticNet LR (R^2^ = 0.926, MAE = 0.216, MSE = 0.074, RMSE = 0.273, RAE = 0.265, RSE = 0.073) at the Alor Setar station and (R^2^ = 0.938, MAE = 0.103, MSE = 0.022, RMSE = 0.150, RAE = 0.218, RSE = 0.061) at the Kota Bharu station. Also, as shown in Table 5, the LSTM model performed better than XGB model (R^2^ = 0.913, MAE = 0.224, MSE = 0.086, RMSE = 0.294, RAE = 0.276, RSE = 0.089) at the Alor Setar station and (R^2^ = 0.937, MAE = 0.112, MSE = 0.023, RMSE = 0.152, RAE = 0.237, RSE = 0.062) at the Kota Bharu station. With the three machine learning models, predicted values pertaining to monthly Ep have been plotted versus the measured values for both stations as displayed in Figs. 8 and 9, respectively. The lower-level pertaining to scatter plot as well as a better fit with regards to the predicted data with that of the observed values in the 1:1 line are the obvious indicators hinting the superiority with regards to the LSTM model over the other two models.

**Table 5:** Statistical results (testing period) of the three machine learning models for predicting monthly Ep under 10 input combinations of meteorological variables for Alor Setar and Kota Bharu.

Station/model	R^2^	MAE	MSE	RMSE	RAE	RSE
**Alor Setar**						
ElasticNet LR-1	0.615	0.483	0.374	0.612	0.610	0.384
ElasticNet LR-2	0.759	0.387	0.237	0.486	0.484	0.240
ElasticNet LR-3	0.849	0.304	0.149	0.387	0.378	0.150
ElasticNet LR-4	0.845	0.296	0.144	0.380	0.366	0.144
ElasticNet LR-5	0.863	0.296	0.136	0.369	0.366	0.136
ElasticNet LR-6	0.734	0.410	0.261	0.511	0.514	0.265
ElasticNet LR-7	0.792	0.361	0.206	0.454	0.448	0.207
ElasticNet LR-8	0.810	0.320	0.159	0.399	0.396	0.159
ElasticNet LR-9	0.862	0.299	0.137	0.371	0.369	0.137
ElasticNet LR-10	**0.926**	**0.216**	**0.074**	**0.273**	**0.265**	**0.073**
XGB-1	0.666	0.439	0.325	0.57	0.555	0.333
XGB-2	0.762	0.372	0.233	0.483	0.466	0.237
XGB-3	0.824	0.325	0.174	0.417	0.403	0.175
XGB-4	0.838	0.309	0.161	0.401	0.383	0.162
XGB-5	0.845	0.309	0.154	0.393	0.383	0.154
XGB-6	0.766	0.380	0.230	0.479	0.476	0.233
XGB-7	0.786	0.358	0.213	0.461	0.445	0.213
XGB-8	0.833	0.327	0.166	0.408	0.404	0.166
XGB-9	0.812	0.349	0.187	0.433	0.431	0.187
XGB-10	**0.913**	**0.224**	**0.086**	**0.294**	**0.276**	**0.089**
LSTM-1	0.741	0.359	0.232	0.482	0.474	0.258
LSTM-2	0.766	0.364	0.212	0.461	0.475	0.233
LSTM-3	0.894	0.249	0.097	0.311	0.323	0.105
LSTM-4	0.925	0.196	0.068	0.261	0.254	0.074
LSTM-5	0.947	0.178	0.048	0.219	0.230	0.052
LSTM-6	0.807	0.341	0.175	0.418	0.446	0.192
LSTM-7	0.884	0.252	0.106	0.326	0.326	0.115
LSTM-8	0.914	0.232	0.079	0.281	0.300	0.085
LSTM-9	0.959	0.150	0.037	0.194	0.194	0.041
LSTM-10	**0.970**	**0.135**	**0.027**	**0.166**	**0.173**	**0.029**
**Kota Bharu**						
ElasticNet LR-1	0.537	0.335	0.175	0.418	0.701	0.462
ElasticNet LR-2	0.721	0.256	0.105	0.325	0.536	0.278
ElasticNet LR-3	0.796	0.211	0.073	0.270	0.453	0.203
ElasticNet LR-4	0.867	0.165	0.048	0.219	0.354	0.133
ElasticNet LR-5	0.883	0.152	0.042	0.205	0.327	0.116
ElasticNet LR-6	0.821	0.197	0.067	0.260	0.414	0.179
ElasticNet LR-7	0.827	0.192	0.062	0.249	0.414	0.172
ElasticNet LR-8	0.904	0.139	0.034	0.186	0.297	0.095
ElasticNet LR-9	0.923	0.114	0.028	0.167	0.243	0.076
ElasticNet LR-10	**0.938**	**0.103**	**0.022**	**0.150**	**0.218**	**0.061**
XGB-1	0.550	0.330	0.170	0.412	0.691	0.449
XGB-2	0.745	0.240	0.096	0.310	0.503	0.254
XGB-3	0.803	0.211	0.070	0.265	0.453	0.196
XGB-4	0.774	0.228	0.082	0.286	0.489	0.225
XGB-5	0.849	0.179	0.054	0.233	0.384	0.150
XGB-6	0.833	0.190	0.063	0.251	0.399	0.166
XGB-7	0.819	0.198	0.065	0.255	0.425	0.180
XGB-8	0.906	0.142	0.033	0.184	0.304	0.093
XGB-9	0.917	0.131	0.030	0.174	0.279	0.082
XGB-10	**0.937**	**0.112**	**0.023**	**0.152**	**0.237**	**0.062**
LSTM-1	0.586	0.335	0.173	0.416	0.677	0.413
LSTM-2	0.796	0.225	0.085	0.292	0.455	0.203
LSTM-3	0.879	0.164	0.048	0.219	0.341	0.120
LSTM-4	0.869	0.177	0.052	0.229	0.366	0.130
LSTM-5	0.915	0.142	0.034	0.184	0.295	0.084
LSTM-6	0.823	0.213	0.074	0.272	0.431	0.176
LSTM-7	0.849	0.195	0.060	0.245	0.405	0.150
LSTM-8	0.906	0.161	0.037	0.194	0.333	0.093
LSTM-9	0.948	0.111	0.021	0.145	0.228	0.051
LSTM-10	**0.986**	**0.058**	**0.005**	**0.074**	**0.120**	**0.013**

As observed in Table 5, the best prediction accuracy was achieved with the models that used the complete meteorological dataset (T_min_, T_max_, S_w_, R_s_, Ep, RH) for both stations, versus other incomplete data input combinations. This established that the accuracy of the model prediction was enhanced with more input parameters in general, which was in line with the earlier studies^3,31^. Also, four input parameters that only lack of R_s_ or S_w_ were sufficient to get acceptable accuracy for monthly Ep estimation. This showed that for monthly Ep estimation in the studied region, relative humidity can be regarded as being more significant versus solar radiation and wind speed. In cases where the only available data are the average temperature data, it was seen that the machine learning models’ prediction accuracy was not sufficient even for the LSTM model (R^2^ = 0.741, MSE = 0.232, MAE = 0.359, RAE = 0.474, RSE = 0.258, RMSE = 0.482) at both the Kota Bharu station and Alor Setar station (R^2^ = 0.586, MSE = 0.173, MAE = 0.335, RSE = 0.413, RMSE = 0.416, RAE = 0.677). This suggested that the machine learning models’ prediction accuracy may not get enhanced with the powerful capabilities pertaining to artificial intelligence, when there are limited meteorological inputs. Better prediction accuracy was achieved, when minimum and maximum temperatures data were available, versus only average temperature as input. In addition, the prediction accuracy of monthly Ep was found to enhance with the implementation of RH or R_s_ versus the models that incorporated only minimum and maximum temperatures as inputs.

Besides, in all ML models, employing Ep as an input slightly was seen to enhance the prediction accuracy. However, with the input combination of T_max_, T_min_, R_s_, S_w_ and RH, it was seen that the statistical values pertaining to machine learning models were near when compared with complete meteorological inputs (i.e., employing Ep as an input). This clearly indicated that the generally estimated monthly Ep values via machine learning models were in good agreement with the measured monthly Ep values. Also, the LSTM model was seen to outperform all other ML models like XGB and ElasticNet LR. This could be due to the architecture pertaining to LSTM neural network that can identify as well as account for the nonlinear behaviour associated with evaporation. The results showed that the LSTM model’s superiority versus other ML models strongly implies that the LSTM model could be employed for predicting monthly pan evaporation with high accuracy. Furthermore, as stated earlier, the application of the proposed methodology to various regions throughout Malaysia using different data sets will also be investigated with the aim of developing a reliable generalised model to predict evaporation.

### Comparison of empirical and machine learning models under the same input combinations

Table 6 shows the performances pertaining to the two empirical models that predict monthly Ep, which have been further compared versus their corresponding machine learning models by employing same input combinations for Kota Bharu and Alor Setar weather stations. Under the input combination of T_a_ and R_s_, the radiation-based model (Stephens and Stewart) provided the lowest prediction accuracy for both stations compared to all ML models under the same input combinations (R^2^ = 0.522, MAE = 0.535, MSE = 0.458, RMSE = 0.677, RAE = 0.681, RSE = 0.477) at the Alor Setar station and (R^2^ = 0.599, MAE = 0.330, MSE = 0.190, RMSE = 0.436, RAE = 0.603, RSE = 0.400) at the Kota Bharu station. On the other hand, the LSTM-6 model outperformed the other empirical and ML models and was able to obtained the highest level of prediction accuracy (R^2^ = 0.807, MAE = 0.341, MSE = 0.175, RMSE = 0.418, RAE = 0.446, RSE = 0.192) at the Alor Setar station and (R^2^ = 0.823, MAE = 0.213, MSE = 0.074, RMSE = 0.272, RAE = 0.431, RSE = 0.176) at the Kota Bharu station. In this context, although the XGB-6 slightly outperformed the LSTM-6 at Kota-Bharu station, it is obvious that this is a single case as the LSTM model is more consistent and could provide higher accuracy compared to empirical and other ML approaches under all different input combinations at both stations.

**Table 6:** Statistical results of the empirical and machine learning models under the same input combination for Alor Setar and Kota Bharu weather stations.

Input combination	Station/model	R^2^	MAE	MSE	RMSE	RAE	RSE
**Alor Setar Station**							
T_a_, R_s_	Stephens and Stewart	0.522	0.535	0.458	0.677	0.681	0.477
	ElasticNet LR-6	0.734	0.410	0.261	0.511	0.514	0.265
	XGB-6	0.766	0.380	0.230	0.479	0.476	0.233
	LSTM-6	0.807	0.341	0.175	0.418	0.446	0.192
T_a_	Thornthwaite	0.303	0.635	0.670	0.819	0.811	0.696
	ElasticNet LR-1	0.615	0.483	0.374	0.612	0.610	0.384
	XGB-1	0.666	0.439	0.325	0.570	0.555	0.333
	LSTM-1	0.741	0.359	0.232	0.482	0.474	0.258
**Kota Bharu Station**							
T_a_, R_s_	Stephens and Stewart	0.599	0.330	0.190	0.436	0.603	0.400
	ElasticNet LR-6	0.821	0.197	0.067	0.260	0.414	0.179
	XGB-6	0.833	0.190	0.063	0.251	0.399	0.166
	LSTM-6	0.823	0.213	0.074	0.272	0.431	0.176
T_a_	Thornthwaite	0.401	0.449	0.330	0.574	0.820	0.693
	ElasticNet LR-1	0.537	0.335	0.175	0.418	0.701	0.462
	XGB-1	0.550	0.330	0.170	0.412	0.691	0.449
	LSTM-1	0.586	0.335	0.173	0.416	0.677	0.413

While, under the input of T_a_ only, the temperature-based model (Thornthwaite) had also much lower accuracy than the corresponding machine learning models with the performance measures as (R^2^ = 0.303, MAE = 0.635, MSE = 0.670, RMSE = 0.819, RAE = 0.811, RSE = 0.696) at the Alor Setar station and (R^2^ = 0.401, MAE = 0.449, MSE = 0.330, RMSE = 0.574, RAE = 0.820, RSE = 0.693) at the Kota Bharu station. On the other hand, the LSTM-1 model was predominant among the empirical and all machine learning models in both stations with the performance measures as (R^2^ = 0.741, MAE = 0.359, MSE = 0.232, RMSE = 0.482, RAE = 0.474, RSE = 0.258) at the Alor Setar station and (R^2^ = 0.568, MAE = 0.335, MSE = 0.173, RMSE = 0.416, RAE = 0.677, RSE = 0.413) at the Kota Bharu station. It is obvious from the statistical results shown in Table 6 that ML models outperformed empirical models and can significantly improve the accuracy of monthly Ep prediction even with the same inputs parameters relying on its superior capabilities to perform complex tasks.

## Conclusion

The present study is aimed at evaluating the machine learning ML technique’s ability for predicting monthly Ep pertaining to two regions in Malaysia. To predict monthly evaporation, we developed three different machine learning models included Extreme Gradient Boosting, ElasticNet Linear Regression and Long Short-Term Memory, along with 10 input combinations pertaining to meteorological variables. The prediction models were tested and trained using available monthly Ep data from 2000 to 2019. The models were trained on 80% of the data and tested on 20%. The models’ accuracies were compared by accounting for standard statistical measures. The prediction accuracy pertaining to monthly Ep differed considerably relies on both the input combination and the model type. The best prediction accuracy was achieved with the models that were using complete meteorological dataset (T_min_, T_max_, R_s_, RH, S_w_, Ep) for both stations, versus other incomplete data input combinations. Four input parameters that lacked just S_w_ or R_s_ were found to be sufficient for the estimation of monthly Ep providing acceptable accuracy. This suggests relative humidity to be more significant when compared with solar radiation and wind speed with regards to monthly Ep estimation pertaining to the studied region. The prediction accuracy was found to be better with the available minimum and maximum temperatures data, versus with those with just the average temperature as input. Moreover, it was found that using Ep as an input slightly improve the prediction accuracy in all ML models. Comparisons were also made between developed ML models and two empirical models, one of which is radiation-based model (Stephens and Stewart) and the other is temperature-based model (Thornthwaite). It was found that the three developed ML models outperformed empirical models with the same input combinations. The performance evaluation revealed that the Long Short-Term Memory provided the most accurate monthly Ep estimates among the empirical models and other machine learning models for both Alor Setar and Kota Bharu stations. The LSTM-10 model statistical performance measures were, R^2^ = 0.970, MAE = 0.135, MSE = 0.027, RMSE = 0.166, RAE = 0.173, RSE = 0.029 for Alor Setar and R^2^ = 0.986, MAE = 0.058, MSE = 0.005, RMSE = 0.074, RAE = 0.120, RSE = 0.013 for Kota Bharu. The results showed that the LSTM model’s superiority versus other ML models strongly imply that the LSTM model could be employed for predicting monthly pan evaporation with high accuracy. Furthermore, the application of the proposed methodology to various regions throughout Malaysia using different data sets will also be investigated with the aim of developing a reliable generalised model to predict evaporation.
